# Risk Factors and Prevalence of Dilated Cardiomyopathy in Sub-Saharan Africa: A Systematic Review

**DOI:** 10.5334/gh.1166

**Published:** 2022-10-21

**Authors:** Lulu S. Fundikira, P. Chillo, R. Mutagaywa, A. Kamuhabwa, G. Kwesigabo, F. W. Asselbergs, L. W. van Laake

**Affiliations:** 1 Muhimbili University of Health and Allied Sciences, P O BOX 65001, Dar es Salaam, Tanzania; 2 Department of Cardiology, Division Heart & Lungs, University Medical Center Utrecht, Utrecht University, 3584 CX Utrecht, the Netherlands; 3 Institute of Cardiovascular Science, University College London, 222 Euston Road, London, NW1 2DA, UK; 4 Health Data Research UK and Institute of Health Informatics, University College London, London, United Kingdom

**Keywords:** Dilated cardiomyopathy, cardiovascular risk factors, sub-Saharan Africa

## Abstract

**Highlights**

- Prevalence of DCM varies widely in SSA.
- Cardiovascular risk factors are important in patients with DCM.
- The role of genetics in idiopathic DCM is not studied in major part of SSA.

## Introduction

### Background

An interesting phenomenon is unfolding in Sub Saharan Africa (SSA) due to globalization and urbanization. The region traditionally plagued with infectious diseases is currently facing a double burden of disease, as evidenced by the rise of non-communicable diseases, mainly cardiovascular diseases (CVDs) [1]. The increase in CVD incidence has resulted in a growing burden of heart failure in SSA [2], a trend that is expected to increase over time [3]. Cardiomyopathies, which are diseases affecting mainly the heart muscles, are a common cause of heart failure worldwide, and represent a significant cause of morbidity and mortality. In 2010, cardiomyopathies were estimated to cause mortality in up to 5.9 of 100,000 individuals globally, and most likely are underdiagnosed [4,5]. In SSA, a contemporaneous systematic review and meta-analysis of the etiology of heart failure performed by Agbor et al. [6] showed that cardiomyopathies (all forms) constituted 21.4% (18.2%–40.2%) of all heart failure (HF) cases, second only to hypertensive heart disease as a cause of heart failure. Among the different types of cardiomyopathies, dilated cardiomyopathy (DCM) is by far the most common SSA [7,8,9].

According to the European Society of Cardiology (ESC), DCM is defined by presence of left or biventricular dilatation and contractile dysfunction in the absence of abnormal loading conditions (such as hypertension or valve disease) or coronary artery disease that is sufficient to cause global contractile impairment [10]. The etiology of DCM is diverse and heterogeneous, including genetic mutations, infections, and autoimmunity, although in most instances the etiology cannot be completely identified [11]. The ESC classifies DCM as familial or non-familial, in which familial cases usually have a genetic cause [10]. However, the American Heart Association classifies DCM as genetic, acquired, or mixed [12]. While idiopathic DCM is mostly genetic, acquired DCM has been associated with the presence of non-modifiable cardiovascular risk factors such as family history, age, ethnicity, and sex, as well as modifiable risk factors such as hypertension, diabetes, tobacco use, physical inactivity, poor nutrition, excessive alcohol consumption, high cholesterol, and obesity, increasing the probability of developing heart failure [13,14].

A look into studies on cardiomyopathies done in Africa from the 2000s onwards shows DCM to be a disease probably representing a final common expression of myocardial damage that could be provoked by multiple insults, including hemodynamic, infective, immunologic, toxic, nutritional, and genetic factors. Idiopathic DCM is defined as a clinical syndrome of heart failure that is associated with impaired systolic function and left ventricular dilatation in the absence of an identifiable cause [9]. In this systematic review we will use these terms in this context.

However, prevalence of DCM among heart failure patients has not been systematically studied in the region, and the relative contribution of the different risk factors to the etiology of DCM is not known in SSA. With the increasing recognition of DCM as a heterogeneous and diverse disease [10,12,15], it is important to understand the contribution of the different cardiovascular risk factors to the clinical presentation of DCM. Identifying risk factors may bring about insightful management consequences, including medical counseling directed to patients and their relatives to avoid or manage the modifiable risk factors so as to halt or prolong the course of DCM.

The advances in sequencing techniques and rigorous clinical work up has allowed for better understanding of genetic basis of idiopathic DCM and precise medicine application to reduce morbidity and prolong life [16]. This review systematically studied the available data published from 2000 onward to capture the current situation in SSA with regard to the prevalence of DCM in patients with heart failure.

## Methods

### Inclusion Criteria

All full-text articles from observational studies (cross-sectional, cohort, retrospective, or prospective) that met the search criteria, published in the English language from January 1, 2000 to December 31, 2020, were included in this review.

### Exclusion Criteria

This review excluded case reports, editorials, comments or expert opinions, as well as letters of study subjects due to lack of peer review. In addition, articles published in a language other than English were excluded. Qualitative studies were also excluded. Protocol for this systematic review was previously published [17].

### Search Strategy

A limited search of PubMed was performed to identify relevant keywords contained in the title, abstract, and subject descriptors. The initial search terms were ‘heart failure,’ ‘cardiomyopathy,’ and ‘sub-Saharan Africa’; these search terms and their synonyms were then used in an extensive search in PubMed. This search was applied to answer question 1 on prevalence. Thereafter, a search was performed to answer question 2 using the terms ‘heart failure,’ ‘cardiomyopathy,’ and the risk factors of interest, which are age, gender, ethnicity, family history, hypertension, diabetes, tobacco use, physical inactivity, poor nutrition, excessive alcohol consumption, high cholesterol, and obesity, in ‘sub-Saharan Africa.’ Filters were added to narrow down to articles published from 2000 on, and in the English language. Indexed articles in PubMed and Embase were included. Taking into account that some journals in Africa may not be indexed in PubMed, searches in Google Scholar was also performed, and the first 300 articles were included. The detailed search terms followed the PICO (Patient/Population/Problem, Intervention/Prognostic Factor, Comparison, Outcome) as per published protocol.

### Risk of Bias and Study Quality

Identified studies that met the inclusion criteria were assessed independently for methodological validity by two reviewers prior to inclusion in the final analysis using Quality Assessment Tool for Observational Cohort and Cross-Sectional Studies found at https://www.nhlbi.nih.gov/health-topics/study-quality-assessment-tools.

### Data Collection

Full copies of articles identified by the search that met the inclusion criteria based on their title, abstract, and subject descriptors were obtained for data synthesis. The collected data was organized in Endnote reference manager and subsequently uploaded to the Rayyan web app for systematic reviews to allow for adequate sorting. Two reviewers independently selected articles against the inclusion criteria. Discrepancies in reviewer selections were resolved by a third author (arbitrator) prior to the selected articles being retrieved. A data extraction tool was developed specifically for quantitative research data extraction based on the work of the Cochrane Collaboration and the Centre for Reviews and Dissemination, as seen published protocol. Two reviewers independently performed data extraction. In cases of missing data, the corresponding authors of the study were approached once by email. Data sorting flow is shown in Annex 1.

### Data Synthesis

The Preferred Reporting Items for Systematic Reviews and Meta-Analyses (PRISMA) guidelines for reporting systematic reviews were applied [18]. A flow diagram is provided to illustrate the literature search and article selection process (annex 1). Furthermore, a map of sub-Saharan Africa with colors to show idiopathic DCM prevalence in each country is included (annex 2). For categorical and continuous variables, mean, median and interquartile range were used to summarise data.

## Results

We included 24 articles with data from 16 SSA countries. All articles were from hospital-based studies, and the majority were prospective studies (10, 41.7%); retrospective and cross sectional studies were each at 7 (29.2%), seen in Table 1. The majority of the studies were graded as fair 14 (58.3%), followed by good quality 10 (41.7%); one article was graded as poor quality (Table 1).

**Table 1:** General aspects of the studies on HF patients in SSA.


VARIABLE	NUMBER OF STUDIES	VALUE

Mean Age, mean(SD)	21	52.5(6.2)

Male sex, median(IQR)	21	44.2(33.2–49.5)

DCM prevalence, median(IQR)	14	20.5(9.7–31.7)

Idiopathic DCM prevalence, median(IQR)	11	13.8(11.0–19.6)

Study type, n (%)	24	

Cross sectional		7(29.2%)

Prospective		10(41.7%)

Retrospective		7(29.2%)

Country, n (%)	24	

Nigeria		6(25%)

Tanzania		4(16.7%)

Uganda		2(8.3%)

Ghana		2(8.3%)

Others		9(37.8%)

SSA*		1(4.2%)

Quality assessment of studies, n (%)	24	

Good		8(33.3)

Fair		15(62.5)

Poor		1(4.2)


* This study involved 9 countries in SAA namely Sudan, Ethiopia, Kenya, Uganda, Mozambique, South Africa, Cameroon, Nigeria and Senegal.

Nigeria had the largest number of studies 6 (26.1%), followed by Tanzania 4 (17.4%), Uganda and Ghana with two studies each at 8.7%. However, THESUS-HF study was the biggest heart failure study in SSA with data from 9 countries (Table 1).

We recorded the prevalence of DCM in patients with HF from 14 studies of 20.5% (9.7–31.7). In patients with HF due to various causes, including DCM, hypertension was noted as the most common cardiovascular risk factor at 53.4% (35.9–59.0), and males constituted 44.2% (33.2–49.5). Other important risk factors were obesity 20.65% (11.2–33.5), tobacco use 6.6% (2.5–19.1), and excessive alcohol intake 10% (2.1–16.0). Black ethnicity and positive family history were only recorded in 3 studies each at 97.3% and 4.0%, respectively. Physical inactivity was only recorded in one study done in Kenya, seen in 73% of the study population. In the reviewed articles, no information was available on nutrition status (Table 3).

Prevalence of idiopathic DCM in HF patients was recorded at 13.8% (11.0–19.6) from 11 studies, seen in Table 1. Only one study done in Chad provided value for DCM (22%) and proportion of idiopathic DCM within the studied population of HF patients at 8%. The criteria for diagnosis of idiopathic DCM in each study were provided as in Table 2.

**Table 2:** DCM prevalence in patients with heart failure in Sub-Saharan Africa.


FIRST NAME AUTHOR, YEAR PUBLISHED	COUNTRY	STUDY DESIGN	STUDY SETTING	SAMPLE SIZE	MALE (%)	MEAN AGE (YEARS)	DCM PREVALENCE %	IDIOPATHIC DCM PREVALENCE %	CRITERIA FOR IDIOPATHIC DCM DIAGNOSIS

A G B Amoah, 2000 [37]	Ghana	Cross sectional	Hospital based	572	NR	42.3	NR	16.8	Stated as idiopathic DCM in the article

K Sliwa et al, 2008 [38]	South Africa	Prospective	Hospital based	844	NR	NR	NR	35.0	ESC guidelines

K Karaye, 2008 [39]	Nigeria	Cross sectional	Hospital based	76	55.7	46.9	10.1	NR	

D B Ojji, 2009 [40]	Nigeria	Cross sectional	Hospital based	340	50.9	50.6	NR	13.8	Idiopathic DCM the left ventricle was dilated (with or without dilatation of the other three cardiac chambers) with global systolic and diastolic dysfunctions in subjects with no known cause of heart failure

O Onwuchewka, 2009 [41]	Nigeria	Cross sectional	Hospital based	423	57.2	54.4	7.3	NR	

A Damasceno, 2012 [8]	SSA	Cross sectional	Hospital based	1006	49.2	52.3	NR	18.8	ESC guidelines

GF Kwan, 2013 [42]	Rwanda	Retrospective	Hospital based	138	27.0	35.0	54.0	NR	

D B Ojji, 2013 [43]	Nigeria	Prospective	Hospital based	1515	49.3	49.0	NR	12.0	The diagnosis was that of exclusion in subjects presenting with features of heart failure without any obvious etiological factor

S Okello, 2014 [44]	Uganda	Retrospective	Hospital based	274	30.3	52.0	31.4	NR	

A Makubi, 2014 [45]	Tanzania	Prospective	Hospital based	427	45.0	55.0	21.1	NR	

O S Ogah, 2014 [28]	Nigeria	Prospective	Hospital based	452	54.9	56.6	7.5	NR	

T B Abebe, 2016 [46]	Ethiopia	Retrospective	Hospital based	311	23.8	53.5	NR	12.5	Idiopathic DCM when there was no other known cardiac cause and had LVEF <50%

G S Bloomfield, 2016 [47]	Kenya	Prospective case-control study	Hospital based	118	49.0	61.0	19.5	NR	

K O Bonsu, 2017 [48]	Ghana	Retrospective	Hospital based	1488	46.6	60.3	19.9	NR	

J T Hertz, 2017 [49]	Tanzania	Retrospective	Hospital based	294	44.2	62.4	8.5	NR	

J C Mwita, 2017 [29]	Botswana	Cross sectional	Hospital based	193	53.9	54.2	NR	19.6	ESC and study of Soweto Guidelines

S Mmbali, 2017 [50]	Tanzania	Prospective	Hospital based	131	43.5	45.3	32.8	NR	

S Okello, 2018 [51]	Uganda	Prospective	Hospital and community	195	32.0	52.0	20.0	NR	

D Malamba-Les, 2018 [23]	DRC	Retrospective	Hospital based	231	47.0	56	47.6	NR	

M M Baba, 2018 [52]	Nigeria	Prospective	Hospital based	354	36.6	46.9	NR	11.0	Stated as idiopathic DCM in the article

C Nkoke, 2019 [53]	Cameroon	Cross sectional	Hospital based	86	44.5	59.4	NR	9.3	Stated as idiopathic DCM in the article

N Madjirangar, 2019 [54]	Chad	Retrospective	Hospital based	100	52.0	40.2	22.0	8.0	Stated as idiopathic DCM in the article

P Pallangyo, 2020 [55]	Tanzania	Prospective	Hospital based	419	43.4	46.4	27.0	NR	


NR = Not recorded.

**Table 3:** Cardiovascular risk factors in HF patients studied in SSA.


VARIABLE	NUMBER OF STUDIES	VALUE

Age in years, mean (SD)	21	52.5(6.2)

Hypertension %, median(IQR)	23	53.4(35.9–59.0)

Male sex %, median(IQR)	21	44.2(33.2–49.5)

Diabetes Mellitus %, median(IQR)	17	11(1.9–12.8)

Tobacco use %, median(IQR)	10	6.6(2.5–19.1)

Physical inactivity %	1	73%

Poor Nutrition %	0	0

Excessive alcohol intake %, median(IQR)	7	10(2.1–16.0)

High cholesterol/Dyslipidemia %; median(IQR)	4	6.6(2.5–9.1)

Obesity %, median (IQR)	6	20.6(11.2–33.5)

Family history %, mean (SD)	2	4.0(0.9)

Ethnicity(blacks),median(IQR)	3	98.4(94.3–99.6)

BMI, median (IQR)	5	25.1(22.7–26.8)

Low level of education %, median(IQR)	6	34.9(3.1–38)


## Discussion

In this systematic review we summarised data from 24 studies in 16 countries in SSA to determine the prevalence of DCM and associated risk factors in acquired form of the condition. We also looked into idiopathic form of DCM and its contribution to heart failure in SSA.

### Prevalence of dilated cardiomyopathy in SSA

We observed that DCM was an important cause of HF in SSA population, with varied weight across the region observed while comparing a study from Nigeria at (7.3%) and another study in Rwanda at 54% [19,20]. However, our findings differ slightly with a retrospective study done in Morroco in 120 patients previously admitted for HF, which showed a prevalence of 33.3% [21]. This difference could be attributed to the methodology used and the small sample size of the later study.

Furthermore, previous studies in SSA have reported on DCM in HF patients in SSA and attributed it to diverse etiologies [9]. The role of infections, such as the HIV pandemic, in its etiology could not be overlooked; this may set SSA apart from other parts of the world and calls for a customized approach [22].

### Cardiovascular risk factors in HF patients including DCM in SSA

In our population, we noted female predominance in HF patients including DCM, this finding differs from a study done in Democratic Republic of Congo by Malamba-Les et al., which observed male predominance (59%; n = 65; P < .01) [23]. The female predominance in our review may be partly explained by the disproportionately high prevalence of HIV in women in SSA which is known to trigger myocardial insult leading to cardiomyopathy [24].

Hypertension was the most common cardiovascular risk factor in HF, including patients with DCM in our review; this is in agreement with review by Yuyun et al. in 2020 that showed that hypertension is more common in SSA (30%) compared to high income countries. This high prevalence is due to undiagnosed hypertension and therefore lack of adequate treatment and control [25]. This could be explained by inadequate primary and secondary level preventive measures as a result of inefficient health care systems in the region and epidemiological transition attributed to lifestyle changes.

A study from the Southern Pacific showed that most of the modifiable risk factors were interrelated with low social economic status, as this is associated with a diet rich in fats and carbohydrates, which in turn leads to obesity and increases chances of developing hypertension and diabetes [26]. Urbanization may also explain the rapid increase of this phenomenon in SSA with increased sedentary lifestyle causing an epidemiological shift with increased non communicable diseases leading to double burden of diseases.

In our review there were observed varied levels of smoking habits, with the study done in Nigeria showing low prevalence at 3.3%, compared to 39% in Kenya [27,28]. This variability could be explained by social norms of different ethnic societies in SSA, however smoking habits are reported to be on the rise in the region and projected to increase in the future [25]. Mwita et al. recorded diabetes mellitus in 15.5% of heart failure patients as an important modifiable etiological factor [29]. We did not find any data on nutritional deficiency in our review; in a discussion to curb the rise of cardiovascular risk factors Kofi reported that as of 2018, only seven countries in SSA had food-based dietary guidelines (FBDGs), which are vital in combating CVD risk factors including diabetes mellitus and dyslipidemia [3].

### Other risk factors in patients in HF patients including DCM cases in SSA

Among factors that may explain the difference between registries are coexistent infections in SSA with the potential to trigger cardiac insult leading to cardiomyopathy. The burden of HIV has played a role in increased cardiomyopathies in the region; Shaboodien et al. found that 21% of the patients with HIV-related cardiomyopathy had myocarditis, as opposed to patients with idiopathic DCM where there was no evidence of myocarditis [30]. It is reported that females bear a higher burden of HIV compared to males in SSA [24]. Additionally, high incidence of peripartum cardiomyopathy in the region may contribute to the female predominance as seen in a study by Karaye et al. [31]. These findings emphasize the need to apply a regional approach of the condition taking into account the diversity of triggers.

### Idiopathic DCM in HF patients in SSA

We observed that idiopathic DCM plays an important role in HF in SSA. The advances in sequencing technology have elucidated genetics at the core of its etiology. It has also highlighted the importance of family screening in patients with idiopathic DCM to rule out familial form [16]. Nevertheless, in SSA studies on genetics of cardiomyopathies, including DCM is limited to southern region specifically South Africa, while in other parts of SSA its diagnosis is based on laboratory findings and imaging techniques with limited access [32,33]. Shaboodien et al., in a review on genetics of inherited cardiomyopathies in Africa, identified only nine studies in DCM, of which seven were conducted in South Africa, while the other two were from Tunisia and Egypt [34]. A study done in South Africa by Ntusi et al., which looked into clinical characteristics and outcomes of familial and idiopathic DCM in 109 patients, noted that the majority of the study population were young, with male predominance, and clinicaly presented with HF by New York Heart Association(NYHA) class III–IV [35]. Another paper from the same cohort published data on the clinical genetics and noted that, of those with familial disease (n = 29), pedigree analysis was consistent with autosomal dominant inheritance in 72.4%, autosomal recessive inheritance in 17.2% and X-linked recessive inheritance in 10.4% [36].

## Limitations

In order to capture literature of the past 20 years, we had to define dilated cardiomyopathy as it appears in reviewed papers; this is in contrast to current definitions. Morever, while discussing risk factors for DCM, we could not clearly separate them from general risk factors for HF due to other causes.

## Conclusions

DCM is a common condition among patients presenting with heart failure in SSA. Phenotypic expression is usually triggered by environmental factors including infections. Cardiovascular risk factors are important in acquired form.There is scant data on the role of genetics in the region in patients with idiopathic DCM.

## Additional Files

The additional files for this article can be found as follows:

10.5334/gh.1166.s1 Annex 1. Flowchart showing article from searches to sorting in RAYYAN.

10.5334/gh.1166.s2 Annex 2. Idiopathic DCM prevalnce(%) in patients with HF in SSA.
