# Supplementary figures and images for: Risk Factors and Prevalence of Dilated Cardiomyopathy in Sub-Saharan Africa: A Systematic Review
Source: Glob Heart. 2022 Oct 21;17(1):76. doi: 10.5334/gh.1166 (PMC9585983; doi:10.5334/gh.1166)

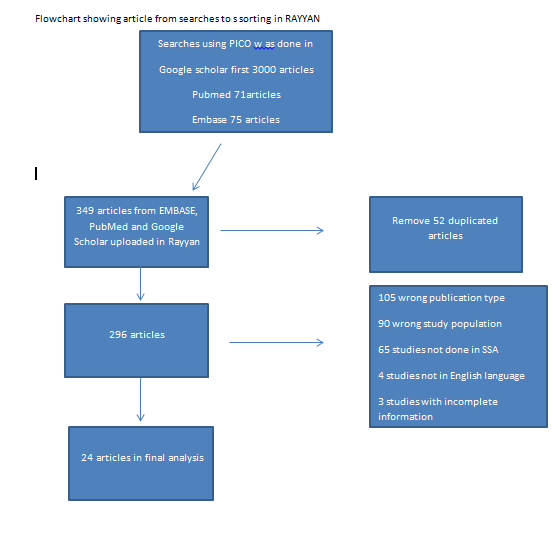

Supplement: Annex 1. — Flowchart showing article from searches to sorting in RAYYAN. [file gh-17-1-1166-s1.png]

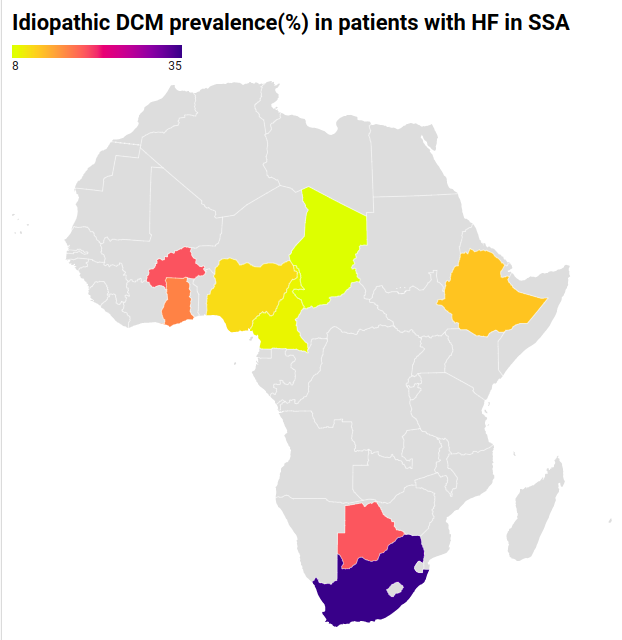

Supplement: Annex 2. — Idiopathic DCM prevalnce(%) in patients with HF in SSA. [file gh-17-1-1166-s2.png]
